# Supplementary material for: Short-Term Mortality in Patients with Heart Failure at the End-of-Life Stages: Hades Study
Source: J Clin Med. 2022 Apr 19;11(9):2280. doi: 10.3390/jcm11092280 (PMC9101156; doi:10.3390/jcm11092280)
Supplement: Supplementary file 1 [file jcm-11-02280-s001.zip › jcm-1664610-supplementary.pdf]

list of collaborators: Gerencia Territorial de Barcelona (Primary healthcare): Muñoz MA, Alonso S, Argemí N, Casademunt M, Furió P, Casajuana E, Torralba N, Verdu JM, Lumillo I, Riesgo J, Alsina, Lara S, Visiedo, Bayo C, Capdevila, Ballester MI, Cuella M, Peralta, Peiró M, Ligero C, Jimenez, Tierz N, Asensio AM.
Institut Universitari d'Investigació en Atenció Primària Jordi Gol (IDIAP Jordi Gol). Navas E.
Departament de Fonaments Clínics-Bioestadística (School of Medicine. Universitat de Barcelona): Abellana R.
Clinica Sant Antoni (Institut medic i de Rehabilitació): Duran J, Sanchez V, Sanchaez A, Breton D, Grasa O.
Bellvitge University Hospital: Calero E
Hospital del Mar Medical Research Institute: Farre N, Ruiz S, Ruiz S, Llinars A, Ruiz P.
Valle Hebron University Hospital: Ferrer R.
